# Spatial patterns of medium and large size mammal assemblages in várzea and terra firme forests, Central Amazonia, Brazil

**DOI:** 10.1371/journal.pone.0198120

**Published:** 2018-05-30

**Authors:** Guilherme Costa Alvarenga, Emiliano Esterci Ramalho, Fabrício Beggiato Baccaro, Daniel Gomes da Rocha, Jefferson Ferreira-Ferreira, Paulo Estefano Dineli Bobrowiec

**Affiliations:** 1 Programa de Pós-graduação em Ecologia, Instituto Nacional de Pesquisas da Amazônia (INPA), Manaus, Amazonas, Brazil; 2 Instituto de Desenvolvimento Sustentável Mamirauá (IDSM), Tefé, Amazonas, Brazil; 3 Departamento de Biologia, Universidade Federal do Amazonas (UFAM), Manaus, Amazonas, Brazil; 4 Graduate Group in Ecology, Department of Wildlife, Fish, and Conservation Biology, University of California, Davis, CA, United States of America; 5 Ecosystem Dynamics Observatory, Instituto de Geociências e Ciências Exatas, Universidade Estadual Paulista (Unesp), Rio Claro, São Paulo, Brazil; Liverpool John Moores University, UNITED KINGDOM

## Abstract

Várzea forests account for 17% of the Amazon basin and endure an annual inundation that can reach 14 m deep during 6–8 months. This flood pulse in combination with topography directly influences the várzea vegetation cover. Assemblages of several taxa differ significantly between unflooded *terra firme* and flooded várzea forests, but little is known about the distribution of medium and large sized terrestrial mammals in várzea habitats. Therefore, our goal was to understand how those habitats influence mammalian species distribution during the dry season. Specifically, we: (1) compared the species composition between a terra firme (Amanã Sustainable Development Reserve) and a várzea forest (Mamirauá Sustainable Development Reserve); and (2) tested the influence of the várzea habitat classes on the number of records, occurrence and species composition of mammalian assemblages. The sampling design in each reserve consisted of 50 baited camera trap stations, with an overall sampling effort of 5015 camera trap days. We used Non-Metric Multidimension Scaling (NMDS) to compare species composition between terra firme and várzea forests, and used Generalized Linear Models (GLM) to assess how habitat types and a habitat diversity index affect mammal distributions. We recorded 21 medium and large sized mammalian species, including 20 species in terra firme and only six in várzea (3443 records). Flood pulse and isolation in várzea forest drove the dissimilarity between these two forest types. In várzea forest, medium size mammals, in general, avoided habitats associated with long flooding periods, while jaguars (*Panthera onca*) appeared to prefer aquatic/terrestrial transition zones. Habitats that remain dry for longer periods showed more mammalian occurrence, suggesting that dispersion via soil is important even for semi-arboreal species. This is the first study to evaluate differential use of várzea habitats by terrestrial mammalian assemblages.

## Introduction

The Amazon rainforest is the world's largest continuous rainforest, covering an area of 7 million hectares and sheltering 51 species of medium and large sized terrestrial mammals in Brazil alone [1]. Despite its extent, the Amazon forest is increasingly threatened by different anthropogenic pressures [2–4]. In this immense forest, the different soil types associated with the variety of lentic and lotic environments form a mosaic of landscapes dominated by upland forests (hereafter ‘terra firme’ forests) surrounded by diverse floodable habitats [5–7]. Often, this contrast among environments acts as an environmental filter for dispersion and establishment of species [8]. Moreover, flooded forests are the most threatened environments in the Amazon basin, suffering a variety of anthropogenic pressures, such as pollution, overharvesting, deforestation for pasture-based farming and hydroelectric dam constructions [4,9,10]. The historical distribution of humans in the Amazon forest is closely related with the great rivers. These areas historically provide resources for housing, cultivation, fishing and hunting [11–13]. Therefore, understanding how these factors influence mammalian species distribution in the landscape is crucial for defining effective conservation areas [14].

Seasonally flooded environments fringing white-water rivers (locally and scientifically known as várzea forests) cover an area of approximately 300,000 km² of the Amazon basin [15]. These rivers have sediment-rich waters with high concentrations of nutrients derived from Andean foothills. Anually, during flood periods, those sediments are deposited on várzea soils [6,16], driving plant community structures and diversity patterns [6,17,18]. On the other hand, terra firme forests rarely flood and, therefore, have lower annual nutrient inputs into the soil [19]. The differences in várzea and terra firme forest productivity and its relationship to the composition of the flora also influence the distribution and structure of the animal species assemblages [20–22].

The difference in mammalian species assemblages between seasonally-flooded forests and terra firme has been reported for several taxa, including bats [23,24], primates [25–27] and medium and large sized mammals [22,28,29]. For exclusively terrestrial species, seasonal flooding is a limiting factor as it decreases the available land area. A number of studies have found seasonal movements of species between várzea and contiguous terra firme forests, in which during the low-water season species migrate to várzea in search of food, such as fruits, seeds and shoots, returning to terra firme when inundation commences [24,28–30].

Locally, topography also influences movement and habitat use of terrestrial mammals [31,32]. During floods, higher areas may form islands in the várzea which can be used as feeding and resting places, especially by species with good swimming capacity or semi-arboreal species that may move between islands [28,29]. During the dry season, species distribution might be influenced by vegetation structure and plant species composition of the different várzea habitats [33,34]. However, little is still known about how habitat classes could influence terrestrial mammal distributions. Only two studies evaluated differential use of várzea habitats by terrestrial mammals, the former with arboreal mammals [35–37] and the latter with a semi-arboreal species, which found a preference by jaguars (*Panthera onca*) for swamp habitats, locally known as Chavascal [38].

The present study aimed to understand how habitat classes influence composition and distribution of medium and large sized terrestrial mammals during low-water season in terra-firme and várzea forests. Specifically, we: (1) compared the similarity of the mammalian assemblage in a continuous terra firme forest and a várzea forest isolated between two large rivers (Amazonas and Japurá), and (2) evaluated how mammalian assemblages responded to different várzea habitat classes. We predicted that mammalian assemblage compositions in the várzea forest will be a subset of the terra firme forest diversity, as just a few number of species are capable of crossing large rivers and to adapt to a flooded forest. We also expected that várzea species will be recorded more frequently in forests flooded for shorter periods (high várzea).

## Materials and methods

### Study area

**Fig 1:**
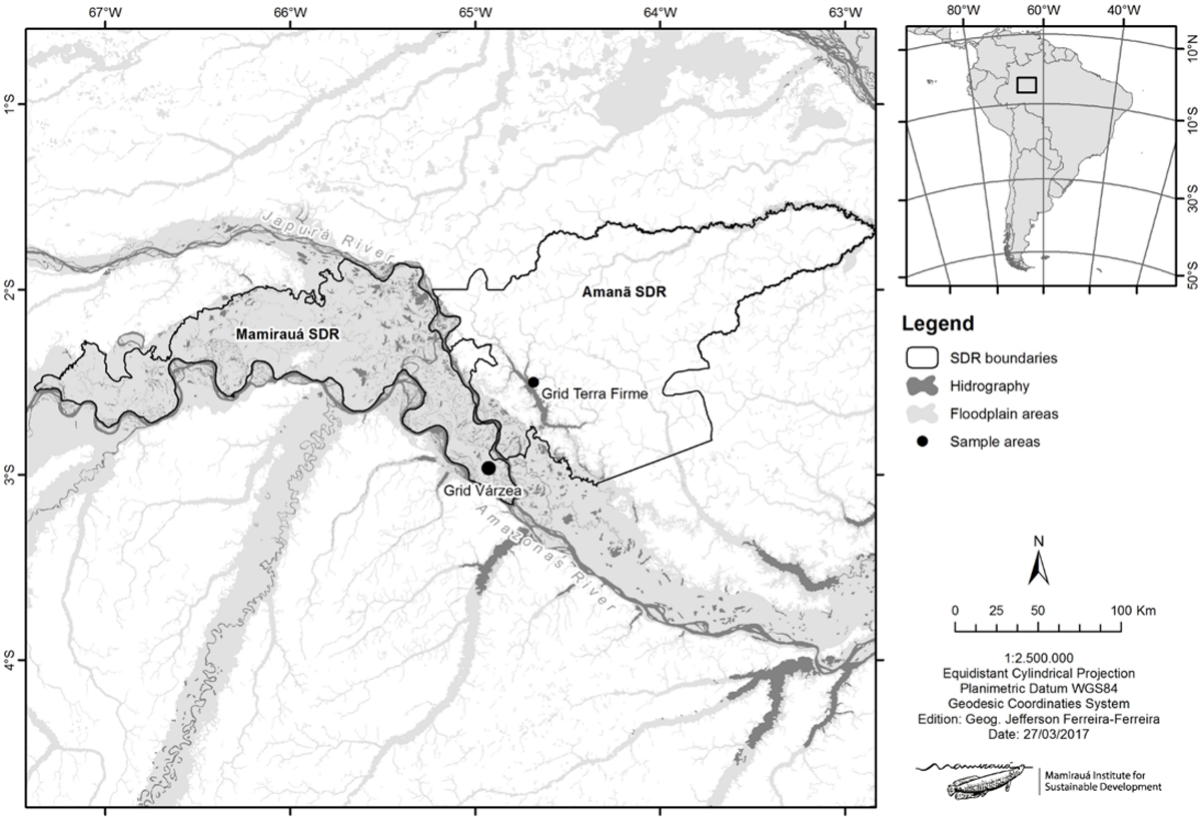
Map of the study areas in Mamirauá Sustainable Development Reserve (MSDR) and Amanã SDR, Central Amazonia, Brazil.

Fieldwork was carried out in Mamirauá (MSDR) and Amanã (ASDR) Sustainable Development Reserves, both located in Central Amazonia, Amazonas State, Brazil (Fig 1). The climate in the region is tropical humid, with an average temperature of 29.5°C and 2373 mm rainfall [33]. The driest period occurs between July and October, and the wettest period between December and March [33].

MSDR (1°49' to 3°09' S, 64°45' to 67°23' W) is delimited by the Amazonas and the Japurá rivers and encompasses an area of 1,124,000 ha entirely composed of várzea forests, being the largest area devoted exclusively to protecting várzea forests in the Amazon. Seasons are divided between flooded (May to July) and non-flooded (September to November) periods, interleaved with the rise (November to May) and fall (July to September) of the waters [39]. ASDR (2°21’S, 64°16’W) lies in the interfluve between the Japurá and Negro Rivers and covers an area of 2,350,000 ha. Along with Jaú National Park and MSDR, the three protected areas form the Central Amazon Corridor, a conservation zone of 5,746,000 ha. The ASDR is mainly composed of unflooded terra firme forests, so that annual floods are limited to the banks of narrower floodplains [40].

### Ethics statement

The Mamirauá Institute for Sustainable Development (MISD) granted research permission for both Reserves. None ethical approval was required as this study did not involve animal handling, nor did it interfere with the animals’ natural behavior.

### Camera trapping

Sampling was carried out during the low water periods in 2013 and 2014. The locations of the camera trap stations were chosen to form a sampling grid with cameras positioned approximately 2 km from each other. Fieldwork in MSDR, for both years, occurred between September and December (80 days) in the southeast region of the reserve. For each sampling, 51 camera trap stations were deployed, distributed over an area of 216.5 km², and totaling a sampling effort of 2040 camera trap days. For logistic reasons, sampling was divided into two consecutive and continuous blocks, each one installed for 40 days. The first block had 26 camera trap stations and the second 25 stations. The distance between stations varied from 1.1 to 5.5 km (2.3 ± 0.26 km, mean ± SD). Each station consisted of two camera traps (model PC800 HyperFire, Reconyx Inc, Holmen, Wisconsin, USA) installed 25 cm from the ground and arranged to face each other, with a 4 m seperation.

Sampling at ASDR, in 2013 and 2014, occurred between December and April (83 days). For each sampling, 50 camera trap stations were deployed, distributed over an area of 130.8 km², and totaling a sample effort of 2075 camera trap days. Sampling followed the same pattern as in MSDR, with two consecutive and continuous blocks each installed for aproximatelly 40 days. Both blocks had 25 camera trap stations and the distance between stations varied from 0.9 to 2.0 km (1.6 ± 0.22 km, mean ± SD). The set up of each camera trap station was the same described for MSDR. The data and metadata of the species sampled are in S1 Table.

The camera traps were configured to record species 24 h/day, with no delay between consecutive triggers and 10 photos (one per second) per trigger. Detections of one species at the same camera trap station in intervals of at least 30 minutes were considered independent. As our study was part of a bigger one focused on the *P*. *onca* population dynamic, at each station between the two camera traps was placed a sardine and egg bait (~ 200 ml) inside a vented container, and fixed to the ground. For logistic reasons these baits were renewed at 14 days intervals. In the second year of sampling, 13 camera trap stations on the second block were not baited for approximately 30 days. To evaluate possible mammalian sampling bias due to bait attraction, we compared the total number of species, the total number of records and the number of records of each species between the non-baited (13) and baited camera trap stations (11) using a permutation *t*-test with 9999 randomizations (S2 Table). There was no difference between treatments and, therefore, records from all stations were used in analysis. To analyze the patterns of distribution, we used a subset of all photographic records including only medium and large sized terrestrial mammals (with average body mass > 1 kg), hence excluding small rodents and primates.

### Várzea landscape analysis

Habitat mapping was performed during a previous study of the várzea forest of MSDR through Synthetic Aperture Radar (SAR) remote sensing [41]. The authors used L-band SAR images (23.6 cm wavelength, 12.5 m spatial resolution) from a PALSAR sensor on board the ALOS satellite, operated by the Japan Aerospace Exploration Agency (JAXA). A multitemporal set of 13 images, acquired from 2007 to 2011, encompassing the southeastern region of MSDR were used in this analysis. SAR systems have the well known ability to capture flooding under canopies, due to the expected increase of returned signals in the presence of water under the vegetation. To better characterize vegetation types, three temporal composite images were produced, enabling vegetation communities to be defined as a combination of vegetation structure and inundation dynamics: temporal average backscattering (TAB), comprising the average backscattering of the entire image series; temporal standard deviation (TSD), comprising the per-pixel standard deviation for all observed values in the series; and lowest water level backscattering (LWB), simply defined as the scene with the lowest observed water stage level. The method consisted of segmenting the image into homogeneous groups of pixels (objects) using these three descriptors, ideally corresponding to homogeneous vegetation classes. This is a region-merging algorithm that begins with a single pixel and a pairwise comparison with its neighbors, with the goal of minimizing the resulting calculated heterogeneity.

After segmentation, the mean and standard deviation backscattering were computed for each image object, separately for all 15 available layers (single date images plus TAB and TSD). Using vegetation type information from 86 survey plots provided by the Mamirauá Institute for Sustainable Development, and supported by Rapid Eye, SPOT-5 and Google Earth TM high resolution imagery, 360 objects were previously classified and selected as training samples (72 objects per class) for subsequent radiometric analysis and classification, based on a multi-sensor interpretation.

The random forests algorithm was used to discriminate the defined classes, which is an ensemble learning method based on classification and regression trees. Instead of a single decision tree, a ‘‘forest” (i.e., ensemble) of individual trees is built through randomization of the training data. Final class predictions are based on using a majority voting scheme (consensus) among the trees in the ensemble, improving predictive accuracy. Finally, the accuracy of the habitat map was assessed using 142 validation points, randomly distributed within the study area, reaching an overall accuracy of 83% [39].

The sampling area was divided into five classes defined by the previous classification: (1) Permanent Water, (2) Soil/Herbaceous Vegetation, (3) Chavascal, (4) Low Várzea and (5) High Várzea. The class Permanent Water represents permanently free water surfaces such as rivers, channels and lakes present even in the driest periods. Soil/Herbaceous Vegetation refers to transient environments dominated mostly by undergrowth and exposed substrate present on the margins of water bodies during periods when the water is low. The last three classes (Chavascal, Low Várzea and High Várzea) are forest formations typical of the várzea. Chavascal is associated with low-lying, prolonged water-logged soils, and has a low canopy dominated by lianas, treelets and shrubs, tolerating floods of 180–240 days/year (water heights varying between 5 and 7 m). Low Várzea comprises arboreal species adapted to flood durations of 120–180 days/year and water level ranging from 2.5 to 5 m. High Várzea tolerates flood durations between 60 and 120 days/year (water level ranging from 1 to 2.5 m), and shares around 30% of tree species with terra firme forests [33,42].

**Fig 2:**
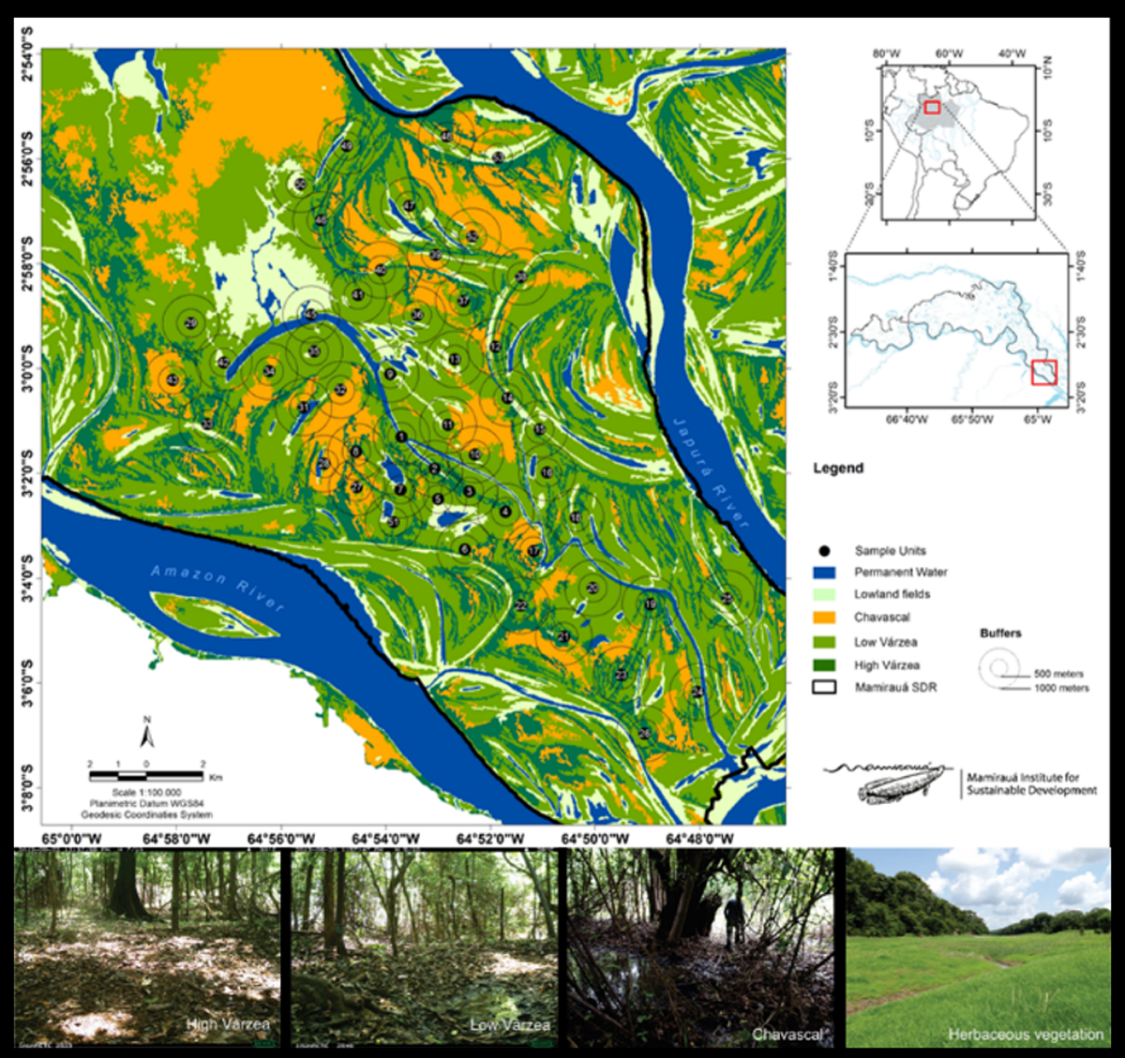
Distribution of the 51 camera trap stations and the respective buffers in both scales, 500 m and 1000 m, in the Mamirauá Sustainable Development Reserve. Each color represent a habitat class of várzea forest.

To determine the influence of habitat classes on the distribution of mammals in the várzea forest, we calculated the occupied area (km²) of each várzea classes at two scales (buffers of 500 and 1000 m radius) around each camera trap station (Fig 2). Due to the lack of knowledge of the mammalian home range in várzea forests, we chose the two buffer sizes to capture the possible effect of scale on mammal species with different body size [43–45]. To find the best response scale, both buffers were tested against all response variables. All Geographic Information System (GIS) procedures were conducted using version 2.12.3 of the QGIS program [46].

### Statistical analysis

All statistical analyses were performed in R, version 3.3.0 [47]. Since the regional flood pulse (height and duration of flood) was highly correlated across the two sampling years (Pearson r² = 0.96) and camera trap stations were in the same location, we pooled the species records of the two sampling years. To analyze if mammalian assemblages were similar between várzea and terra firme, we reduced the matrix dimensionality of the medium and large sized mammalian species recorded using Non-Metric Multidimensional Scaling (NMDS) based on the Bray-Curtis dissimilarity index using the 'vegan' package [48]. We standardized the camera trap stations weight by dividing the number of records of each species by its total number of records, and then for the camera trap station total of records (wisconsin function, MARGIN = 1). This reduces the weight of stations with many records in the ordination analysis. Subsequently, we compared the assemblages of várzea and terra firme using a Permutational Multivariate Analysis of Variance (PERMANOVA, adonis function, ‘vegan’ package) based on the Bray-Curtis Index with 9999 permutations [49,50]. We compared the number of records per sampling effort (camera trap days) and total number of mammal records per station in várzea and terra firme using a Student *t*-test.

To understand the effect of várzea habitats on mammals distribution, we first tested for multicollinearity between predictor variables (areas covered by Permanent Water, Soil/Herbaceous Vegetation, Chavascal, Low Várzea, High Várzea and Shannon index) using a Pearson correlation. Low Várzea vegetation was correlated with Chavascal (buffer 500 m: r = -0.64; buffer 1000 m: r = -0.71), therefore Low Várzea was excluded from the analysis. The Shannon index (H') was calculated using the area (km²) occupied by each class in the buffers of 500 and 1000 m in each camera trap station (diversity function, 'vegan' package). High values represented greater heterogeneity in landscape and greater equitability, while low values indicate dominance of an individual habitat class. As response variables, we used number of species, number of records, species composition and number of records of each mammalian species at each camera trap station. We used one-axis NMDS solution (Bray-Curtis dissimilarity index) with presence and absence data as the mammalian species composition. For single-species analysis, we only considered species that occur in more than 25% of the camera trap stations (five species).

We also tested for spatial autocorrelation of residuals of the multiple regression response variables using Moran's I index on SAM V.4 software [51]. To perform Moran’s I index, nine distance classes with equal numbers of connections were used. The upper limits of the distances classes were 1.99, 3.60, 4.82, 5.00, 7.11, 8.30, 9.72, 11.57, 13.57 and 19.06 km. The significance of each Moran's I value was tested with 9999 randomizations. Moran's I values for the nine distance classes were all between -0.19 and +0.12 for all response variables (five species, number of records, number of species and species composition), indicating that there was no spatial correction in the mammal occurrence data.

To assess the influence of habitat classes (Permanent Water, Soil/Herbaceous Vegetation, Chavascal and High Várzea) and Shannon index on mammal distribution (five species, total number of records, total number of species and species composition) we used Generalized Linear Models (GLM). We chose Gaussian distributions (for NMDS values–first axis), Poisson (for count data), Quasipoisson and Negative Binomial (for overdispersed count data, S3 Table), according to distribution frequency of the response variable data in histograms [52]. For species with records in less than 50% of camera trap stations (*Coendou prehensilis* and *Nasua nasua*), we used Zero Inflation models from the 'pscl' R package [53,54]. The Zero Inflation model separate the data into two sets, (1) values equal to zero and (2) values larger than zero. The excess of zeros is analyzed with a GLM Binomial that calculated the probability of finding false- and true-zeros. The model then analyzes values larger than zeros for Poisson distributions (ZIP). The assumptions of GLMs and Zeros Inflated model were assessed by plotting residuals in relation to predicted values and quantile-quantile plots (QQ-plot). All predictor variables of GLMs and Zero Inflated model were standardized to a mean of zero and a standard deviation of one prior to analysis to facilitate comparison of their relative effects. Due to high values we had three outliers, two of porcupine (*Coendou prehensilis*) and one of coati (*Nasua nasua*). We decided to exclude them; however, analysis with and without those outliers produced the same results.

## Results

### Comparisons between várzea and terra firme forests

**Fig 3:**
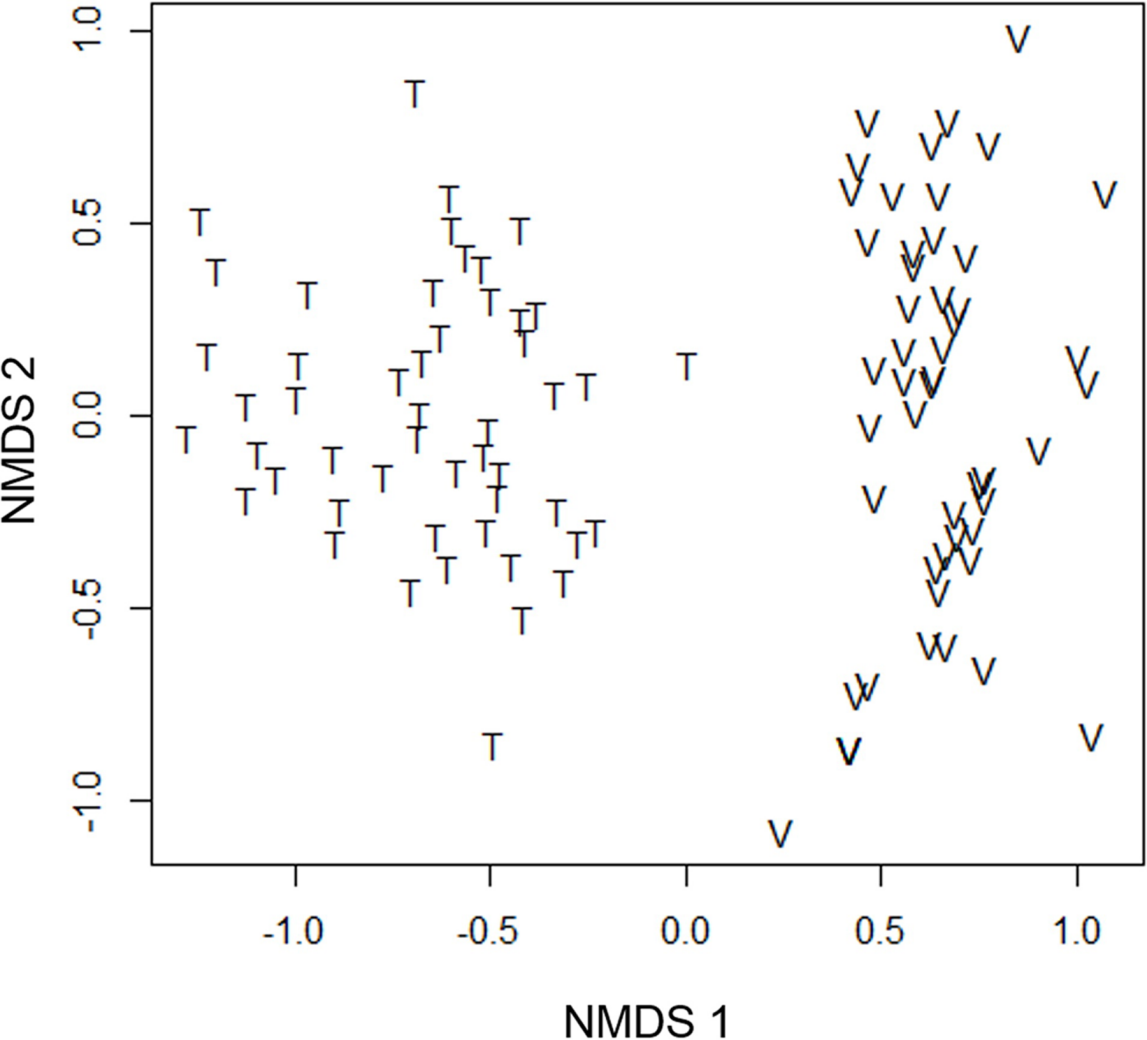
Non-metric multidimensional scaling ordination of the medium and large mammal species composition in terra firme (T) of Amanã Sustainable Development Reserve (ASDR) and várzea (V) of Mamirauá SDR, Central Amazonia, Brazil.

**Fig 4:**
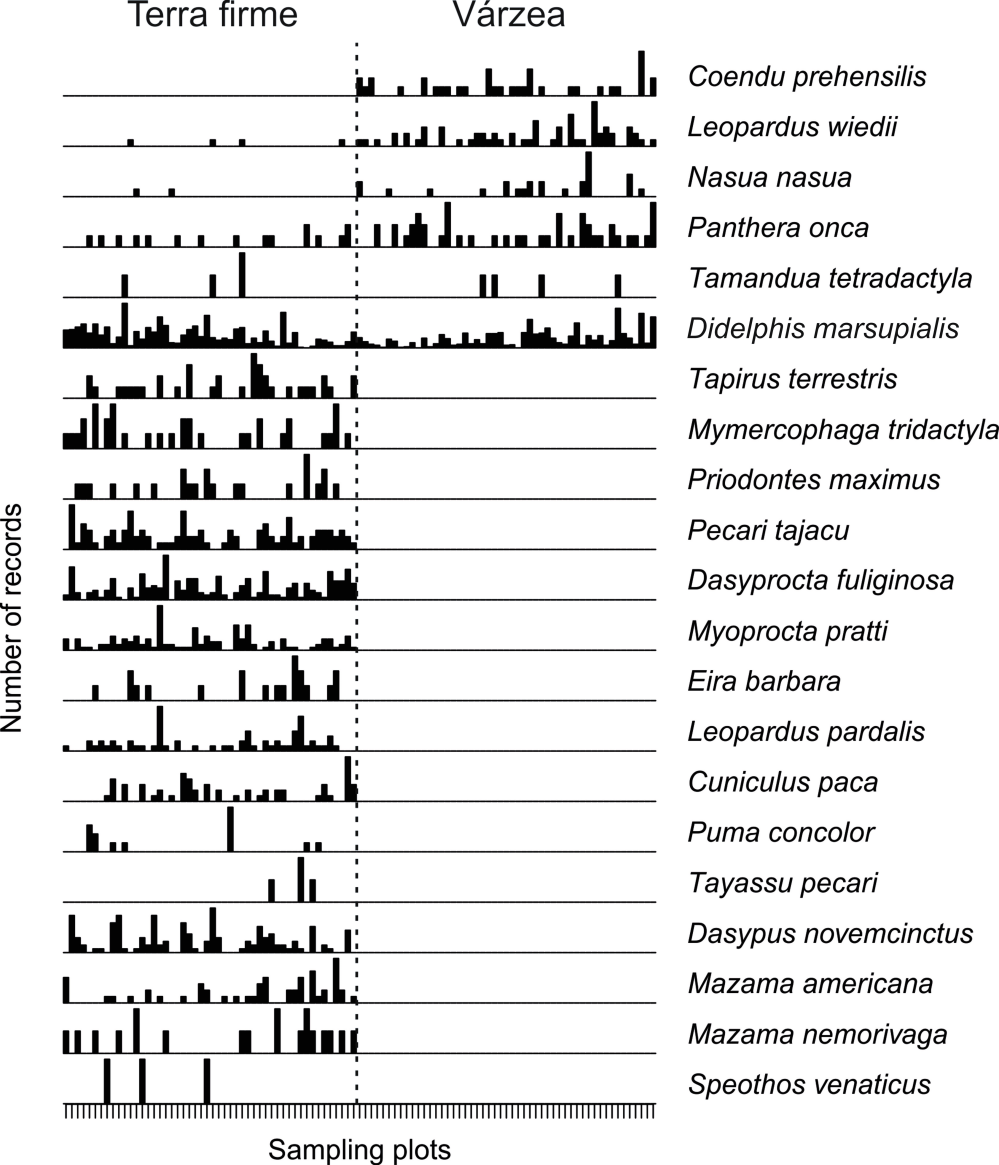
Distribution of the medium and large mammal species records in 101 camera trap stations installed in terra firme forest of Amanã Sustainable Development Reserve (ASDR) and várzea forest of Mamirauá SDR. The dotted line divides the two forest types.

We obtained 1154 medium and large mammal records in the MSDR várzea and 2289 records in the ASDR terra firme forest. The number of records per effort in terra firme was twice (1.20 record/traps*day) that of várzea forest (0.56 record/traps*day) (t = 5.97, P < 0.001). We recorded 21 species, six in várzea and 20 in terra firme (t = 18.97, P < 0.001) (S4 Table). Among the 21 species recorded, five occurred in both forest types, 15 species were unique to terra firme and only *C*. *prehensilis* exclusively recorded in várzea. Among the few species found in várzea, *P*. *onca*, margay (*Leopardus wiedii*) and *N*. *nasua* were recorded more frequently in várzea than in terra firme. The ordination of camera trap stations along the two axes of NMDS explained 72% of the variation in species composition (stress = 0.18). We observed a marked difference between composition of medium and large mammal species for várzea and terra firme forests per camera trap station (PERMANOVA, R² = 0.33, P < 0.001), mainly in axis 1 (Fig 3). At the regional scale, the várzea mammal assemblage represented a subgroup of terra firme forests mammals (Fig 4).

### Effect of habitat classes on distribution of species in várzea forests

As shown by Shannon index (H'), buffer zone habitat class coverage varied greatly between camera trap stations. Within the 500 m buffer, Low Várzea was the dominant habitat, occupying an average of 0.44 ± 0.18 km² (mean ± SD), followed by Chavascal (0.12 ± 0.16 km²) and High Várzea (0.13 ± 0.13 Km²). This pattern was repeated for the 1000 m buffer (Table 1). There were two camera trap stations within the dominion of Low Várzea (H' < 0.05) in the 500 m buffer. On the other hand, low dominance and high habitat diversity (H' > 1.0) was exhibited for 17 stations in the 500 m buffer and 36 stations in the 1000 m buffer.

**Table 1:** Area of coverage (km^2^) of the five habitat classes in várzea of Mamirauá Sustainable Development Reserve, Central Amazonia, and the Shannon index in the 500 m and 1000 m scales buffers around the camera trap stations. Data are presented as mean ± standard deviation and between parenthesis minimum and maximum values.

Predictor variables	500 m buffer scale	1000 m buffer scale
Water	0.03 ± 0.05 (0–0.18)	0.12 ± 0.13 (0–0.59)
Soil/Herbaceous	0.07 ± 0.08 (0–0.39)	0.25 ± 0.17 (0–0.68)
Chavascal	0.12 ± 0.16 (0–0.56)	0.55 ± 0.53 (0–1.93)
Low Várzea	0.44 ± 0.18 (0–0.78)	1.69 ± 0.57 (0.48–2.97)
High Várzea	0.13 ± 0.13 (0–0.48)	0.51 ± 0.34 (0–1.47)
Shannon Index	0.87 ± 0.31 (0–1.44)	1.06 ± 0.25 (0.23–1.47)

**Fig 5:**
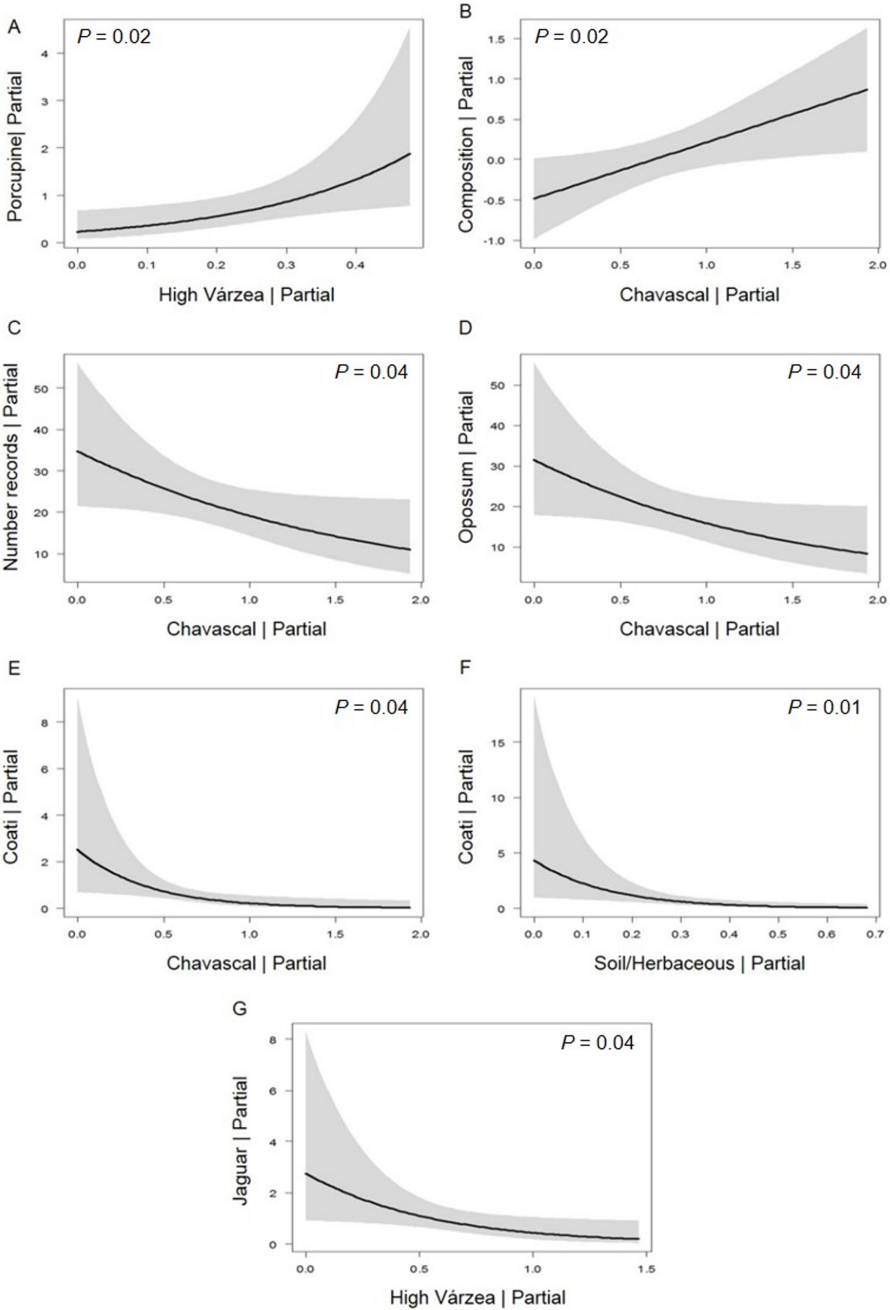
Partial regressions of the response variables with significant relationship to the habitat classes in várzea forest of Mamirauá Sustainable Development Reserve, Central Amazonia, Brazil. (A) Porcupine (*Coendou prehensilis*)–High Várzea; (B) Composition–Chavascal; (C) Number of Records–Chavascal; (D) Opossum (*Didelphis marsupialis*)–Chavascal; (E) Coati (*Nasua nasua*)–Chavascal; (F) Coati (*N*. *nasua*)–Soil/Herbaceous; (G) Jaguar (*Panthera onca*)–High Várzea.

In general, with the exception of *P*. *onca*, mammal species avoided the habitats most susceptible to flooding. In the 500 m buffer scale, *C*. *prehensilis* was the only species positively related to High Várzea (Fig 5A). In the 1000 m buffer, the composition of the mammalian assemblage was influenced by Chavascal (Fig 5B). The total number of records and opossum species (*Didelphis marsupialis*) were negatively associated with Chavascal (Fig 5C and 5D). Similarly, the number of *N*. *nasua* records was lower in areas associated with water bodies, Soil/Herbaceous Vegetation and Chavascal (Fig 5E and 5F). On the other hand, the number of *P*. *onca* records was lowest in High Várzea forest. (Fig 5G).

### Discussion

Our results show that mammalian assemblages in terra firme and várzea are different at the local scale, and that mammalian composition of the várzea forest is a subset of the terra firme forest. Both results suggest a limited effect of overall species migration between habitats during dry-season. Sampling in Mamirauá várzea was undertaken in the lower Japurá River, where its course is approximately 2 km wide and flows into the Amazonas River. For this reason, the Mamirauá várzea remains isolated during the dry season, and even species that are strong swimmers, like jaguars (*P*. *onca*), avoid crossing the river, either from várzea to terra firme, or terra firme to várzea (E. E. Ramalho personal communication). All species recorded in várzea were semi-arboreal and able to survive in the forest canopy during flooded months. These results suggest that flood pulse, along with the isolation of Mamirauá reserve, act as environmental filters selecting species able to survive in such a large area of várzea forest.

### Number of species

A previous study at Amanã and Mamirauá reported six species not found in our sampling. However, this study used other sampling methods, such as interviews with local residents, direct and indirect sighting and shooting. Of the six species, four (*Hydrochaeris hydrochaeris*, *Lontra longicaudis*, *Potos flavus*, and *Pteronura brasiliensis*) were recorded in both terra firme and várzea, while two others (*Puma concolor* and *Tayassu pecari*) were recorded only in várzea [55]. The first four species are semi-aquatic or arboreal, and are difficult to record with camera traps restricted to the understory [56,57]. The last two were registered only occasionally in more than ten years of camera trap surveys at Mamirauá (E. E. Ramalho personal communication), suggesting that records may not be from resident populations. It is common to record more species when complementary sampling methods are used, which underscores the importance of using a variety of techniques when conducting fauna surveys [58–60]. However, the total number of species and the composition of medium and large terrestrial mammal assemblages recorded at Amanã and Mamirauá SDRs were similar to those described in other camera trap-based studies in Amazon forest [59,61–64].

### Spatial patterning in mammalian assemblages

Our results show that mammals from várzea are a subset of the species found in terra firme, confirming both our initial hypothesis and the pattern described in the literature for a number of other taxa [22,24,65–67]. The várzea forest assemblage was composed of semi-arboreal mammals. The same species distribution pattern was described previously using other sampling techniques [55,68].

The most plausible explanation for differences in várzea and terra firme mammalian assemblage composition is linked to seasonal flooding and the isolation of the Mamirauá várzea by two large rivers, Amazonas and Japurá. The seasonality of várzea flooding explains spatial differences for several taxa, especially during the flooding season when the two environments have the greatest contrast [24,30]. However, our sampling was performed during the unflooded season, suggesting that other flood pulse-associated factors may contribute to the differences between environments. The Amazonas and Japurá rivers probably act as a barrier to fauna, separating the Mamirauá várzea from adjacent mainland forests, producing a pattern similar to that found for primates [69] and terrestrial mammals [22] in other regions of the Amazon basin. Even though taxa such as carnivores, perissodactyls and artiodactyls are known to be strong swimmers, it has been reported that relatively narrow rivers (~50 m) can act as barriers to terrestrial mammals in the lower Purus River [22]. Therefore, it is not unreasonable to propose that mammalian species avoid crossing the Japurá and Amazonas rivers (both slightly >2 km wide) during dry season months.

The greatest number of records of terrestrial species in terra firme also indicates the presence of a larger number of individuals and, consequently, may lead to a higher total biomass, a pattern that also has been reported in the lower Purus River [22]. The lack of a flood pulse in terra firme makes it possible for terrestrial mammals to live there throughout the year. Strictly terrestrial species such as *Tapirus terrestris*, *Pecari tajacu*, *T*. *pecari*, *Mazama americana* and *Mazama nemorivaga* recorded only in terra firme have a larger body size compared to semi-arboreal species of the várzea forest, which contributes to the high animal biomass that terra firme forests can support.

### Effects of habitat on distribution of mammal species in várzea forest

All response variables were scale-dependent, showing relationships with only one buffer size. Our results match previous studies in finding a scale-related effect in species response [70–74]. This pattern is attributed to variations in the coverage of landscape elements associated with the size of the buffer, as well as species intrinsic factors, such as home range size, ability to move through different landscape types, environmental requirements and life histories [72,75,76]. Therefore, the spatial scale should be taken into account in ecological landscape studies [44,45,77].

The total number of records and assemblage species composition were influenced by the presence and extent of Chavascal vegetation. The lower number of records in Chavascal supports our hypothesis that environments with extended inundation are avoided by várzea-living mammalian species. Factors such as a protracted inundation period (~8 months), and permanently water-logged soils [78] seems to act as an ecological filter even for semi-arboreal mammal species.

The influence of Chavascal was also evident at the species level, as both *D*. *marsupialis* and *N*. *nasua* avoided this habitat. *N*. *nasua* is a gregarious procyonid, with a diet composed mainly of invertebrates and fruits [79,80], while *D*. *marsupialis* is a highly adaptable solitary generalist [81,82]. Both species have scansorial habits, but often use the forest floor to move between trees and for foraging [83–86], and, therefore, tend to avoid permanently waterlogged Chavascal soils. *N*. *nasua* was also negatively associated with exposed soils and open herbaceous vegetation areas. This exclusively várzea habitat is associated with steep banks (known locally as "barrancos") and water body margins [6], indicating that *N*. *nasua* is more sensitive to open areas than other semi-arborial mammals inhabiting várzea. Such avoidance may occur because open environments with no trees that can be used as escape routes are probably less safe from predators.

*C*. *prehensilis* is an arboreal species that in captivity spends 85% of its time in trees [87,88]. However, camera traps were set at ground level, so lack of occurrence for this species in terra-firme forests should be interpreted with caution. Even so, the distribution of *C*. *prehensilis* in High Várzea seems plausible. High Várzea forest has the greatest density and highest number of tree species of all várzea habitats [6,33], which helps to explain why an arboreal species, like *C*. *prehensilis*, was most recorded in this habitat.

The negative association between *P*. *onca* and High Várzea in Mamirauá could be associated with the distribution of the main prey species [38]. During the non-flooded season, *P*. *onca* mainly preys on sloths [36], caiman and their nests, all concentrated in low-lying areas transitional between water and land [89], usually far from the High Várzeas [33]. In Viruá National Park, a tendency for the use of flooded environments by individuals of *P*. *onca* was also observed [90]. Generally, large felines tend to alter their use of space depending on prey availability [91–93]. Spatial use by *L*. *wiedii* could also be associated with prey distribution. However, 21 known *L*. *wiedii* prey species were recorded, ranging from arboreal mammals, birds, lizards and amphibians [94]. This variety of available prey probably allows *L*. *wiedii* to use various várzea habitats in a more homogeneous way and, therefore, no precise habitat association has been found for this species in várzea.

### Conservation implications

Of the 21 species recorded, four (*Myrmecophaga tridactyla*, *Priodontes maximus*, *T*. *terrestris* and *T*. *pecari*) appear as ‘vulnerable’ on the global IUCN Red List [95]. Seven of the recorded species are considered ‘vulnerable’ at the national level: *Leopardus pardalis*, *L*. *wiedii*, *M*. *tridactyla*, *P*. *onca*, *P*. *maximus*, *P*. *concolor* and *Speothos venaticus* [96]. The presence of these species demonstrates the effectiveness of wildlife conservation in large protected areas (hereafter ‘PAs’) in Amazônia [97,98]. Even predators, such as *P*. *onca* in Mamirauá SDR and *L*. *pardalis* in Amanã SDR, that are killed in retaliation for attacks on domestic animals still have stable populations in the reserves [99,100]. However, continued human population growth in sustainable use PAs constitutes a potential threat to game animals [101]. In addition, a significant portion of PAs in the Amazonian biome (~ 42%) are threatened by modifications to existing legislation that will result in changes such as size reduction, diminished restrictions on human activities and the full loss of PA status [3]. Such threats reinforce the urgent need to document wildlife in PAs and access their relationship to diferent habitat types, especially for endangered species, to assist management plan formulation [3].

Our results indicate that várzea habitats influence the distribution of medium and large sized mammals. Our understanding about the use of different várzea habitats by terrestrial mammals needs further focused studies for effective management planning. The low number of species found in várzea does not imply that this habitat should be neglected during conservation planning. On the contrary, a variety of studies have shown that many species use seasonally flooded forests when connected to non-flooded forests, suggesting that a combination of flooded and non-flooded habitats is crucial to the long term maintenance of viable populations [20,21,28,30,57]. The importance of preserving large areas of adjacent várzea and terra firme forests is shown by the nested species distribution pattern between these forests. Due to the effect of the environmental filter caused by the flood pulse and isolation, some feline species (*P*. *concolor* and *L*. *pardalis*) and large mammals (deer, peccaries and tapir) were recorded exclusively in terra firme forests, while other felines (*P*. *onca* and *L*. *wiedii*) were more common in várzea. Várzea forests have been identified as an important habitat for preservation of a diverse range of animal groups, including fish [102], amphibians [65], primates [103,104] and bats [24]. However, várzeas are being constantly and consistently threatened by such human activities as hunting, and creation of areas for grazing cattle and raising crops [11]. Beyond that, in a scenery of climate change, the low resilience of lowland forests and the shifts in wildlife populations make this habitat extremely susceptible to collapse, which would impact the entire Amazon basin [105,106].

## Supporting information

S1 Table Data file. (XLSX)

S2 Table Student *t*-test to evaluate the possible influence of the use of bait in the records of medium and large mammalian species in camera trap stations in várzea of Mamirauá Sustainable Development Reserve, Central Amazonia, Brazil. ‘No bait’ is a comparison of baited and unbaited stations, while ‘Control’ is a comparison of stations with the same treatment.
(XLSX)

S3 Table Best models result for medium and large size mammals in relation to predictor variables in várzea of Mamirauá Sustainable Development Reserve, Central Amazonia, on the 500 m and 1000 m scales. GLM = Generalized Linear Model; ZIP = Zero-Inflated Poisson. GLM distributions were: G = gaussian; P = poisson; QP = quasi-poisson and NB = negative binomial.
(XLSX)

S4 Table Medium and large mammalian species records and the number of camera trap stations with records in várzea and terra firme forests, Central Amazonia, Brazil. (XLSX)
